# Light Conversion upon Photoexcitation of NaBiF_4_:Yb^3+^/Ho^3+^/Ce^3+^ Nanocrystalline Particles

**DOI:** 10.3390/nano13040672

**Published:** 2023-02-09

**Authors:** Enrico Trave, Michele Back, Davide Pollon, Emmanuele Ambrosi, Leonardo Puppulin

**Affiliations:** 1 Department of Molecular Sciences and Nanosystems, Università Ca’ Foscari Venezia, Via Torino 155, 30172 Venice, Italy; 2 WPI Nano Life Science Institute (WPI-NanoLSI), Kanazawa University, Kanazawa 920-1192, Ishikawa, Japan

**Keywords:** nanophosphor, lanthanide ion emission, upconversion luminescence, visible-to-NIR downconversion, chromaticity tuning

## Abstract

NaBiF_4_ nanocrystalline particles were synthesized by means of a facile precipitation synthesis route to explore upconversion emission properties when doped with lanthanide ions. In particular, the incorporation of the Yb^3+^-Ho^3+^-Ce^3+^ triad with controlled ion concentration facilitates near-IR pumping conversion into visible light, with the possibility of color emission tuning depending on Ce^3+^ doping amount. We observed that introducing a Ce^3+^ content up to 20 at.% in NaBiF_4_:Yb^3+^/Ho^3+^, the chromaticity progressively turns from green for the Ce^3+^ undoped system to red. This is due to cross-relaxation mechanisms between Ho^3+^ and Ce^3+^ ions that influence the relative efficiency of the overall upconversion pathways, as discussed on the basis of a theoretical rate equation model. Furthermore, experimental results suggest that the photoexcitation of intra-4f Ho^3+^ transitions with light near the UV-visible edge can promote downconverted Yb^3+^ near-IR emission through quantum cutting triggered by Ho^3+^-Yb^3+^ energy transfer mechanisms. The present study evidences the potentiality of the developed NaBiF_4_ particles for applications that exploit lanthanide-based light frequency conversion and multicolor emission tuning.

## 1. Introduction

Light conversion includes a series of photon management procedures for modifying light energy and frequency in a selected spectral range, which find wide application in diverse technologies such as solar and photovoltaic, photosynthetic biomass production, bioimaging and lighting [1,2,3,4,5]. Trivalent lanthanide ions (Ln^3+^)-doped materials historically represent an interesting class of luminescent light converters since, under appropriate photoexcitation, they give rise to photophysical mechanisms which can lead to wavelength shift and multicolor emission, with careful control of the chromaticity output [6,7,8,9,10,11].

Upconversion (UC), where two or more absorbed low-energy photons are converted into a higher-energy one, and downconversion (DC) via quantum cutting (QC), where the absorption of a single high-energy photon originates two or more photons emitted at lower energies, are widely mentioned as nonlinear processes that bring to light frequency modification [12]. These mechanisms are highly fostered in lanthanide-doped phosphors due to the unique optical properties exhibited by lanthanide ions, deriving from a characteristic ladder-like arrangement of energy levels from ultraviolet to near-infrared and parity-forbidden intra-4f transitions with long-lived intermediate states. Among the most studied types of lanthanide-based UC systems, phosphors based on Yb^3+^-Ln^3+^ (Ln^3+^ = Er^3+^, Ho^3+^, Tm^3+^) pairs still receive great attention due to the incomparable near IR-to-visible UC emission efficiency [8,13,14,15]. This is due to an overall photophysical process based on a sequence of sensitizer–activator energy transfer mechanisms, which exploits the large absorption cross section of Yb^3+^, the sensitizer ion, under 980 nm photoexcitation, and the resonant level matching with the activator ion (Er^3+^, Ho^3+^, Tm^3+^), which is promptly promoted to high-energy states by the conversion of several absorbed near-infrared (NIR) photons and then relaxes radiatively, producing an emission spectrum characteristic of the Ln^3+^ energy levels scheme. In particular, both Yb^3+^-Er^3+^ and Yb^3+^-Ho^3+^ pairs show UC photoluminescence in the visible range dominated by a green and a red emission, both originating from two-photon UC processes. The relative intensity of the two bands determines the chromaticity output and can be controlled by concentration of the doping Ln^3+^ species.

Strategies for UC efficiency enhancement as well as for color output tuning include the incorporation of a further doping species for manipulating the Yb^3+^-Ln^3+^ UC pathways and then sensitize and/or foster specific emission outputs. Relevant results in Yb^3+^-Er^3+^ UC efficiency increase was achieved by embedding alkali or transition metal ions, such as Li^+^, Fe^3+^, Cd^2+^, into Ln^3+^-doped matrix, while pure single red band resulted by means of Mn^2+^ codoping of NaYF_4_:Yb^3+^/Er^3+^ NPs [16,17,18,19,20]. Redshift tuning of UC emission is particularly appealing in the field of bioimaging and biolabeling, since it achieves emission in the first biological window and simultaneous suppression of visible light noise. In this regard, Ce^3+^ incorporation has been reported as a valid method for green-to-red conversion in Yb^3+^/Ho^3+^ codoped fluoride hosts [21,22,23,24,25]. Photophysical mechanisms underlying the sensitizer–activator process are influenced by the presence of Ce^3+^ since it can enhance the population of the activator intermediate states involved in the red band UC emission process by virtue of its peculiar 4f energy level structure.

To develop efficient lanthanide-based UC phosphors, the choice of the host matrix is crucial. It is well known that fluoride materials offer several advantages, such as the extremely low phonon energy. Mainly for this reason, they are widely considered for the preparation of both bulk and nanostructured lanthanide-doped UC systems used in several fields, such as theranostics, nanothermometry, color displays, optical encoding, to name a few [26,27,28,29,30,31]. Bismuth-based compounds are characterized by a high refractive index, which induces a reduction in multiphonon relaxation rates and enhancement of spontaneous emission probability; namely, they are a popular choice as a host for optical dopants suitable for the realization of UC phosphors and optical thermometers [32,33,34,35,36]. Recently, Yb^3+^/Ln^3+^ (Ln^3+^ = Er^3+^, Ho^3+^, Tm^3+^) codoped NaBiF_4_ nanoparticles have been prepared by means of a room-temperature, one-pot synthesis procedure, representing a valuable alternative with respect to conventional methods based on high-temperature solvothermal and hydrothermal route employed for the preparation of fluoride-based particles [37]. Later, the issue of thermal and chemical stability of the bismuth based fluoride materials has been addressed to exploit their potential in possible photonic applications [38].

Within the scope of a research activity related to the development of a specific class of Bi-based fluoride compounds with tailored optical properties, this study was conceived to elucidate the properties of light conversion of Yb^3+^/Ho^3+^/Ce^3+^ tridoped NaBiF_4_ nanocrystalline particles (NPs). The investigation of UC emission chromaticity control as a function of Ce^3+^ content is aimed at establishing the condition for green-to-red conversion tuning. A theoretical model based on a steady-state rate equations scheme for Ho^3+^ energy level population is adopted to account for the different photophysical mechanisms taking place in the Yb^3+^-Ho^3+^ UC pathways, and to define the role of Ce^3+^ in the emission chromaticity shift, finding a real agreement with the observed experimental trend. Furthermore, spectroscopic results show that UV-visible excitation promotes Yb^3+^ NIR emission, which is particularly efficient through photoexcitation of Ho^3+^ energy levels around the UV-visible edge. This may represent a possible fingerprint of a DC process triggered by a Ho^3+^-Yb^3+^ QC mechanism. The point is discussed on the basis of a proper photophysical scheme, considering that the observed visible-to-NIR QC-mediated DC expands the potentiality of the investigated NaBiF_4_:Yb^3+^/Ho^3+^/Ce^3+^ NPs for applications as a spectral converter.

## 2. Materials and Methods

### 2.1. Chemicals

Bi(NO_3_)_3_·5H_2_O (99.99%), NH_4_F (99.9%), Yb(NO_3_)_3_·5H_2_O (99.9%), Ho(NO_3_)_3_·5H_2_O (99.9%), Ce(NO_3_)_3_·5H_2_O (99.9%), NaNO_3_ (99.9%), ethylene glycol (EG, 99.8%), ethanol (EtOH, 99.8%), Milli-Q water. All reagents were purchased from Sigma Aldrich, Italy.

### 2.2. Synthesis

The preparation route of the investigated Ln^3+^-doped NPs is a modification of the procedure reported by Lei et al. in [37]. In a typical synthesis, 1 mmol of Bi, 1 mmol of Ln^3+^ nitrates and 2 mmol of NaNO_3_ were dissolved into 10 mL of EG. In another beaker, 6 mmol of NH_4_F was dissolved in 25 mL of EG, and then added to the above solution under stirring; on the basis of the results of our previous work [38], the NH_4_F amount was chosen to stabilize the hexagonal NaBiF_4_ phase. The obtained solution was stirred, at room temperature, for one minute, and the product was collected by centrifugation and washed with anhydrous ethanol three times before natural drying. The synthesized NaBiF_4_ NPs incorporate Yb^3+^ and Ho^3+^ ions with doping level set to 10 and 2 at.%, respectively, as optimal condition to maximize the UC emission, with different amounts of Ce^3+^ up to 20 at.% for controlled chromaticity output tuning.

### 2.3. Characterization

X-ray powder diffraction (XRPD) measurements were performed by means of a Philips diffractometer with a PW 1319 goniometer with Bragg–Brentano geometry, connected to a highly stabilized generator, a focusing graphite monochromator and a proportional counter with a pulse-height discriminator. Nickel-filtered Cu Ka radiation and a step-by-step technique were employed (steps of 0.05° in 2θ), with collection times of 30 s per step.

Size and morphology determination of the nanoparticles and EDX analysis were carried out with a Carl Zeiss Sigma VP Field Emission Scanning Electron Microscope (FE-SEM) equipped with a Bruker Quantax 200 microanalysis detector.

Room-temperature photoluminescence (PL) and PL excitation (PLE) measurements, as well as time-resolved PL analysis, were carried out by means of a FluoroLog 3–21 system (Horiba Jobin-Yvon) equipped with a 450 W xenon arc lamp as excitation source, whose wavelength was selected by a double Czerny–Turner monochromator and signal detection stage including an iHR300 single grating monochromator coupled to a Hamamtsu photomultiplier tube (model R928P for visible range; model R5509-73 N_2_-cooled for NIR range). Alternatively, PL spectra were acquired by means of a QE65 Pro Ocean Optics spectrometer. Upconversion measurements were performed by using a CNI MDL-III-980 diode laser as 980 nm photon pumping source, with output power of 2 W over a spot of 5 × 8 mm^2^ (power density of 5 W/cm^2^). For time-resolved PL investigation, the Fluorolog system operated in TCSPC/MCS mode, and the sample excitation was achieved through a pulsed laser source (Horiba SpectraLED) featuring 460 nm peak wavelength and 30 nm spectral FWHM.

## 3. Results and Discussion

The following results represent a comprehensive characterization of a Ce^3+^-free Yb^3+^/Ho^3+^ codoped sample (Ce_0) as compared to tridoped samples embedding a Ce^3+^ content of 2 (Ce_2), 5 (Ce_5), 10 (Ce_10) and 20 at.% (Ce_20).

### 3.1. Structural and Morphological Characterization

**Figure 1:**
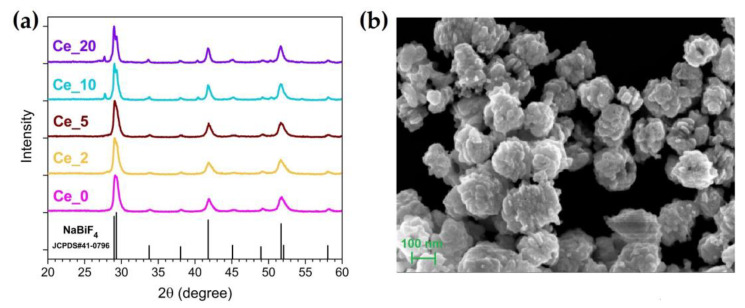
(**a**) XRPD patterns of the Ln^3+^-doped NaBiF_4_ samples with different Ce^3+^ content. (**b**) FE-SEM image of Ce_10 sample.

Figure 1a reports the XRD measurements of the investigated samples. All the diffractograms match well with the crystallographic pattern of the hexagonal NaBiF_4_ phase (chart JCPDS#41-0796), regardless of Ln^3+^ doping content. At the highest Ce^3+^ amount it is noticed the appearance of some faint peaks, which could be attributed to the formation of secondary fluoride phases. From the XRD pattern fitting, we estimated the trend of the cell volume parameter as a function of the Ce^3+^ content. In this regard, Figure S1 shows that the cell volume progressively shrinks by increasing the Ce^3+^ doping level, tending to a plateau for large concentration, in agreement with the fact that Ce^3+^ has a smaller ionic radius with respect to Bi^3+^ (1.14 Å vs. 1.17 Å, respectively).

Morphological properties of the studied NPs can be characterized by the representative SEM image of the tridoped sample with 10 at.% of Ce^3+^ (Ce_10), which is shown in Figure 1b. The synthesized NaBiF_4_ NPs appear as nm-sized spheroidal aggregates, resulting from the assembly of smaller primary nanocrystals [38]. Further SEM images relating to the other investigated samples are included in Figure S2. EDX spectrum together with related elemental maps for Ce_10 sample are reported in Figures S3 and S4, respectively.

### 3.2. UC Properties and Color Tuning Effect

The analysis of the fluorescence properties characterizing the investigated Ln^3+^-doped NPs firstly focuses on UC emission due to Yb^3+^-Ho^3+^ sensitizer–activator coupling and on the chromaticity tuning driven by Ce^3+^ incorporation.

**Figure 2:**
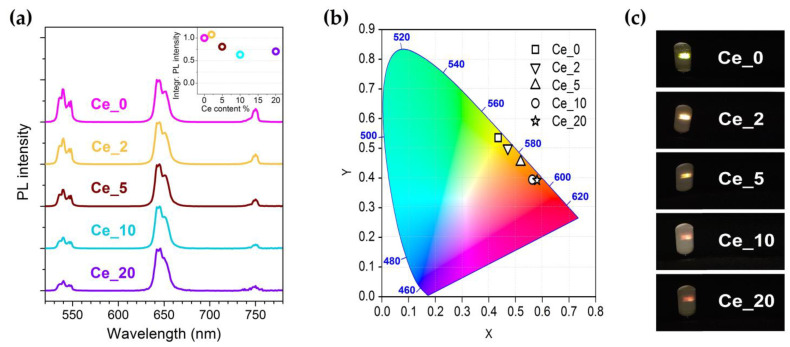
(**a**) UCPL spectra under 980 nm excitation of the Ln^3+^-doped NaBiF_4_ samples with different Ce^3+^ content; each spectrum is normalized to the RED band signal. In the inset, trend of integrated PL intensity of the whole visible UC emission vs. Ce^3+^ content, with signals normalized to Ce_0 sample. (**b**) Chromaticity diagram showing color coordinates tuning for the Ln^3+^-doped NaBiF_4_ samples. (**c**) Digital camera images of the powder samples under 980 nm light exposure.

Figure 2a presents upconversion photoluminescence (UCPL) spectra measured in the visible range under 980 nm light pumping and representative NPs with different Ce^3+^ content. The spectra are dominated by three main emission features with maximum at around 540 nm (named as GRN band, being peaked in the green range), 645 nm (named as RED band, being peaked in the red range) and 750 nm, attributed to Ho^3+ 5^S_2_/^5^F_4_ → ^5^I_8_, ^5^F_5_ → ^5^I_8_ and ^5^S_2_/^5^F_4_ → ^5^I_7_ transitions, respectively. It can be observed that the progressive increase in Ce^3+^ content determines a decrease in intensity for the GRN band compared to the RED one. In addition, for Ce^3+^ content higher than 5%, the overall luminescence emission signal settles down at about 70% of the intensity of the Ce^3+^ undoped sample (Ce_0), as shown in the inset of Figure 2a. This evidence suggests that the impact of any detrimental effects, such as fluorescence quenching due to high lanthanide ions concentration, is rather limited for the system under investigation.

The CIE chromaticity diagram and the photographs of the observed luminescence spots are reported in Figure 2b,c, respectively. The Ce^3+^-induced color rendering index modification is clearly appreciable, going from a pale green emitted spot for the Ce^3+^ undoped sample to dark orange, reaching 20 at.% of Ce^3+^ doping.

**Figure 3:**
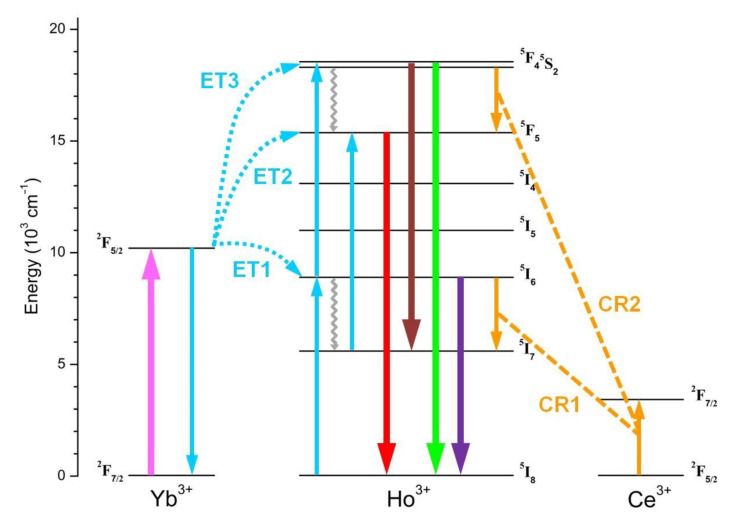
Scheme of the energy level diagram representing UC and transition mechanisms originating the PL emissions observed for the Ln^3+^-doped NaBiF_4_ samples. The pink upward arrow refers to Yb^3+^ ground-state absorption process (GSA); thick downward arrows refer to Ho^3+^ radiative relaxations, where the attributed colors refer to emissions in the red, green and NIR spectral ranges; cyan and orange arrows refer to Yb^3+^-Ho^3+^ energy transfer (ET1, ET2, ET3) and Ce^3+^-mediated cross-relaxation (CR1, CR2) processes; grey wavelike arrows refer to multiphonon relaxations.

In the context of the photophysical mechanisms underlying the UCPL activity observed for the Ln^3+^-doped NPs, the diagram of Figure 3 depicts the main relaxation and transition processes that involve 4f energy levels for the Yb^3+^-Ho^3+^-Ce^3+^ triad. The trigger of the overall mechanism is the intra-4f Yb^3+ 2^F_7/2_ → ^2^F_5/2_ ground-to-excited-state transition under 980 nm light, whose absorption by Yb^3+^ ions is characterized by a very large cross-section. Due to the long Yb^3+ 2^F_5/2_ lifetime, the Ho^3+^ upper excited states can be populated through a very effective Yb^3+^-Ho^3+^ energy transfer (ET) interaction. The diagram highlights the three main mechanisms, labeled as ET1, ET2, and ET3, that lead to the excitation of the Ho^3+ 5^I_6_, ^5^F_5_ and ^5^S_2_/^5^F_4_ levels, respectively, and thus activate the UC emission pathways for the generation of the observed emissions in the visible range.

As previously reported, the GNR band, as well as the emission peaked at 750 nm, is a consequence of the relaxation process from the Ho^3+ 5^S_2_/^5^F_4_ excited state, which is populated through a sequence of ET1 and ET3 mechanisms involving Ho^3+ 5^I_6_ state as the intermediate level. On the other hand, the RED band originates from the relaxation of the Ho^3+ 5^F_5_ state, whose excitation can occur through two possible UC paths. One ends with the direct Ho^3+ 5^F_5_ population through non-radiative relaxation of Ho^3+ 5^S_2_/^5^F_4_ level after a combined sequence of ET1 and ET3 mechanisms. The other is instead based on a sequence of ET1 and ET2 processes, interspersed by Ho^3+ 5^I_6_ non-radiative relaxation to the Ho^3+ 5^I_7_ level. Therefore, both paths include a multiphonon relaxation step which is supposed to strongly affect the overall efficiency for the RED band emission process. In fact, it is worth considering that both ^5^S_2_/^5^F_4_ → ^5^F_5_ and ^5^I_6_ → ^5^I_7_ transitions span an energy gap of about 3000 cm^−1^. Since the typical phonon energy for fluoride-based hosts is relatively low (i.e., in the order of 500 cm^−1^), the upper excited level depopulation must involve a substantial number of phonons, limiting the occupancy of the lower excited level and then the resulting RED band intensity.

In this scenario, Ce^3+^ incorporation becomes effective. As shown in the diagram of Figure 3, Ce^3+^ ion admits a unique intra 4f transition involving the ^2^F_5/2_ ground and the ^2^F_7/2_ excited states. The separation between these energy levels matches the gap that characterizes both the Ho^3+ 5^S_2_/^5^F_4_ → ^5^F_5_ and ^5^I_6_ → ^5^I_7_ transitions. This implies that cross-relaxation (CR) mechanisms take place, involving the transitions Ho^3+ 5^I_6_ + Ce^3+ 2^F_5/2_ → Ho^3+ 5^I_7_ + Ce^3+ 2^F_7/2_ (labeled as CR1) and Ho^3+ 5^S_2_/^5^F_4_ + Ce^3+ 2^F_5/2_ → Ho^3+ 5^F_5_ + Ce^3+ 2^F_7/2_ (labeled as CR2), which flank the multiphonon relaxation steps in the overall upconverting process that leads to the population of the Ho^3+ 5^F_5_ excited state. UCPL measurements reported in Figure 2a demonstrate that the efficiency of the CR1 and CR2 processes is such as to lead to a manifest RED band enhancement with respect to GRN one as the Ce^3+^ content increases.

### 3.3. Rate Equation Modeling of UC Mechanisms

In order to account for the impact of Yb^3+^-Ho^3+^ ET and Ce^3+^-mediated CR processes on the resulting UCPL activity, we have revisited the model proposed by Chen et al. in [21] to formalize a system of rate equations describing the time evolution of the population of Ho^3+^ excited energy levels involved in the observed emission processes:

(1) $$\frac{dN_{1}^{Ho}}{dt}=w_{21}^{MP}N_{2}^{Ho}+c_{2}N_{2}^{Ho}N_{0}^{Ce}-w_{1}N_{1}^{Ho}-k_{1}N_{1}^{Yb}N_{1}^{Ho}\;,$$

(2) $$\frac{dN_{2}^{Ho}}{dt}=k_{0}N_{1}^{Yb}N_{0}^{Ho}-w_{2}N_{2}^{Ho}-k_{2}N_{1}^{Yb}N_{2}^{Ho}-c_{2}N_{2}^{Ho}N_{0}^{Ce}\;,$$

(3) $$\frac{dN_{3}^{Ho}}{dt}=w_{43}^{MP}N_{4}^{Ho}+k_{1}N_{1}^{Yb}N_{1}^{Ho}+c_{4}N_{4}^{Ho}N_{0}^{Ce}-w_{3}N_{3}^{Ho}\;,$$

(4) $$\frac{dN_{4}^{Ho}}{dt}=k_{2}N_{1}^{Yb}N_{2}^{Ho}-w_{4}N_{4}^{Ho}-c_{4}N_{4}^{Ho}N_{0}^{Ce}\;.$$

The symbols used in the equations have the following meaning: $N_{i}^{Ho}$ (with i=0, 1, 2, 3, 4) refers to the population density of Ho^3+ 5^I_8_ ground and ^5^I_7_, ^5^I_6_, ^5^F_5_, ^5^S_2_/^5^F_4_ excited levels, respectively; $N_{i}^{Yb}$ (with i=0, 1) refers to the population density of Yb^3+ 2^F_7/2_ ground and ^2^F_5/2_ excited levels, respectively; $N_{i}^{Ce}$ (with i=0, 1) refers to the population density of Ce^3+ 2^F_5/2_ ground and ^2^F_7/2_ excited levels, respectively; $w_{i}$ (with i=1, 2, 3, 4) refers to the overall transition rate from Ho^3+^ i level to the lower ones, while $w_{21}^{MP}$ and $w_{43}^{MP}$ are the multiphonon-assisted relaxation rates for Ho^3+ 5^I_6_ → ^5^I_7_ and ^5^S_2_/^5^F_4_ → ^5^F_5_ transitions, respectively; $k_{i}$ (with i=0, 1, 2) refers to the coupling constant for the Yb^3+^-mediated ET processes involving Ho^3+ 5^I_8_ → ^5^I_6_, ^5^I_7_ → ^5^F_5_ and ^5^I_6_ → ^5^S_2_/^5^F_4_ transitions, respectively;$\;c_{i}$ (with i=2, 4) refers to the coupling constant for the Ce^3+^-mediated CR processes involving Ho^3+ 5^I_6_ → ^5^I_7_ and ^5^S_2_/^5^F_4_ → ^5^F_5_ transitions, respectively.

In steady-state conditions under cw pumping excitation, from Equations (1)–(4) we obtain the following expressions for the Ho^3+^ excited levels:

(5) $$N_{1}^{Ho}=\frac{\left(w_{21}^{MP}+c_{2}N_{0}^{Ce}\right)k_{2}k_{0}N_{1}^{Yb}N_{0}^{Ho}}{\left(w_{1}+k_{1}N_{1}^{Yb}\right)\left(w_{2}+k_{2}N_{1}^{Yb}+c_{2}N_{0}^{Ce}\right)}\;,$$

(6) $$N_{2}^{Ho}=\frac{k_{0}N_{1}^{Yb}N_{0}^{Ho}}{\left(w_{2}+k_{2}N_{1}^{Yb}+c_{2}N_{0}^{Ce}\right)}\;,$$

(7) $$N_{3}^{Ho}=\frac{\left[k_{1}\left(w_{21}^{MP}+c_{2}N_{0}^{Ce}\right)\left(w_{4}+c_{4}N_{0}^{Ce}\right)+k_{2}\left(w_{43}^{MP}+c_{4}N_{0}^{Ce}\right)\left(w_{1}+k_{1}N_{1}^{Yb}\right)\right]k_{0}{\left(N_{1}^{Yb}\right)}^{2}N_{0}^{Ho}}{w_{3}\left(w_{2}+k_{2}N_{1}^{Yb}+c_{2}N_{0}^{Ce}\right)\left(w_{1}+k_{1}N_{1}^{Yb}\right)\left(w_{4}+c_{4}N_{0}^{Ce}\right)}\;,$$

(8) $$N_{4}^{Ho}=\frac{k_{2}k_{0}{\left(N_{1}^{Yb}\right)}^{2}N_{0}^{Ho}}{\left(w_{2}+k_{2}N_{1}^{Yb}+c_{2}N_{0}^{Ce}\right)\left(w_{4}+c_{4}N_{0}^{Ce}\right)}\;.$$

Assuming that the transition rates for the Ho^3+ 5^I_7_ and ^5^I_6_ levels are much larger than the corresponding upconversion rates, the latter terms can be relaxed in Equations (5)–(8), leading to the following reformulation of the previous expressions:

(9) $$N_{1}^{Ho}=\frac{k_{0}k_{2}\left(w_{21}^{MP}+c_{2}N_{0}^{Ce}\right)N_{0}^{Ho}}{w_{1}\left(w_{2}+c_{2}N_{0}^{Ce}\right)}N_{1}^{Yb}\;,$$

(10) $$N_{2}^{Ho}=\frac{k_{0}N_{0}^{Ho}}{\left(w_{2}+c_{2}N_{0}^{Ce}\right)}N_{1}^{Yb}\;,$$

(11) $$N_{3}^{Ho}=\frac{k_{0}\left[k_{1}\left(w_{21}^{MP}+c_{2}N_{0}^{Ce}\right)\left(w_{4}+c_{4}N_{0}^{Ce}\right)+w_{1}k_{2}\left(w_{43}^{MP}+c_{4}N_{0}^{Ce}\right)\right]N_{0}^{Ho}}{w_{1}w_{3}\left(w_{2}+c_{2}N_{0}^{Ce}\right)\left(w_{4}+c_{4}N_{0}^{Ce}\right)}{\left[N_{1}^{Yb}\right]}^{2}\;,$$

(12) $$N_{4}^{Ho}=\frac{k_{0}k_{2}N_{0}^{Ho}}{\left(w_{2}+c_{2}N_{0}^{Ce}\right)\left(w_{4}+c_{4}N_{0}^{Ce}\right)}{\left[N_{1}^{Yb}\right]}^{2}\;.$$

**Figure 4:**
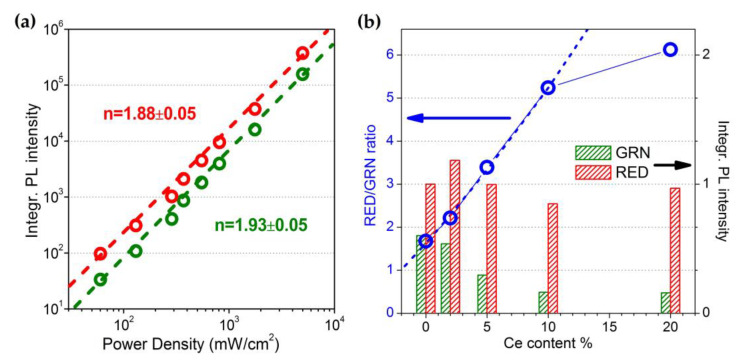
(**a**) Log–log plot of the integrated UCPL intensity of GRN and RED emissions vs. 980 nm pumping power density; the order n of the UC processes corresponds to the slope of the linear fit (dashed lines) of the experimental data; the measurements were performed on Ce_10 sample. (**b**) Bar graphs of GRN and RED emission intensity and scatter + line plot (hollow blue dots and solid blue line) of RED-to-GRN intensity ratio vs. Ce^3+^ content; the dashed line corresponds to a parabolic fit of the intensity ratio, considering the data in Ce^3+^ content range of 0–10 at.%.

Considering that Yb^3+^ light absorption occurs linearly in the adopted pump power regime, it can be stated that the parameter $N_{1}^{Yb}$ describing the population density of the Yb^3+ 2^F_5/2_ excited level is proportional to the intensity of the excitation source. Therefore, Equations (11) and (12) would imply a quadratic pump power dependence for the $N_{3}^{Ho}$ and $N_{4}^{Ho}$ population density parameters, and then for the emission intensitiy of GRN and RED bands, since they originate from Ho^3+ 5^F_5_ and ^5^S_2_/^5^F_4_ excited level relaxations, respectively. As a matter of fact, this behaviour finely agrees with the trends shown in the log–log plot of Figure 4a, where the linear fit of the experimental data related to the power density dependence of UCPL emission for Ce_10 sample resulted in a slope close to 2 for both GRN and RED emissions, thus confirming the occurrence of a two-photon UC process.

For a qualitative assessment of the observed chromaticity tuning effect, the bar graphs in Figure 4b evidence the Ce^3+^ content dependence for the intensity of the GRN and RED bands, as extracted from PL bands shown in the spectra of Figure 2a. Moreover, Figure 4b also reports the trend of the RED-to-GRN intensity ratio as a function of Ce^3+^ content, evidencing a progressive growth of this parameter up to 10 at.% of Ce^3+^, whereas with a Ce^3+^ content of 20 at.%, a sort of saturation effect takes place, leading to a less pronounced increase in the ratio.

From Equations (11) and (12), we can formalize an expression of the Ce^3+^ content dependence for the RED-to-GRN intensity ratio parameter:

(13) $$\begin{array}{cc} \frac{I_{RED}}{I_{GRN}}\;\propto \;\frac{N_{3}^{Ho}}{N_{4}^{Ho}} & =\frac{\left[k_{1}\left(w_{21}^{MP}+c_{2}N_{0}^{Ce}\right)\left(w_{4}+c_{4}N_{0}^{Ce}\right)+w_{1}k_{2}\left(w_{43}^{MP}+c_{4}N_{0}^{Ce}\right)\right]}{w_{1}w_{3}k_{2}}\\ & =\frac{w_{43}^{MP}}{w_{3}}+\frac{w_{21}^{MP}w_{4}k_{1}}{w_{1}w_{3}k_{2}}+\left(\frac{c_{4}}{w_{3}}+\frac{w_{21}^{MP}k_{1}c_{4}}{w_{1}w_{3}k_{2}}+\frac{w_{4}k_{1}c_{2}}{w_{1}w_{3}k_{2}}\right)N_{0}^{Ce}+\frac{k_{1}c_{2}c_{4}}{w_{1}w_{3}k_{2}}{\left[N_{0}^{Ce}\right]}^{2}\;. \end{array}$$

This equation accounts for the observed increase in the intensity ratio with the Ce^3+^ doping level. This behaviour is predictable by observing the structure of Equation (12), where the $N_{4}^{Ho}$ parameter, namely, the GRN emission intensity, is forced to decrease as the Ce^3+^ content rises.

Given the parabolic trend of the expressions in Equation (13), Figure 4b also includes a fit of the ratio values with a parabolic function, which results in a real agreement with the trend of the experimental values up to 10 at.% of Ce^3+^.

Furthermore, to generalize the proposed scenario and to validate the adequacy of the proposed rate-equation model, it is worth pointing out that similar experimental results are reported for Yb^3+^/Ho^3+^/Ce^3+^ tridoped fluoride systems investigated in the works of Gao et al. [22,39,40], Chen et al. [21], and Pilch-Wróbel et al. [41], where the trend of the resulting GRN intensity ratio recalls a parabolic dependence on Ce^3+^ doping level, in some cases reaching possible saturation effects at large Ce^3+^ content.

### 3.4. Efficiency of Ce^3+^-Mediated CR Processes

The observed color tuning effect is closely linked to the Ce^3+^ doping level adopted for the investigated samples. From the rate equations previously reported, it can be inferred that the CR processes impact on the occupancy of the Ho^3+^ levels involved in the overall UCPL mechanism. In particular, the CR1 and CR2 processes (identified by the coupling constants $c_{2}$ and $c_{4}$, respectively) constitute a depletion channel for the Ho^3+ 5^I_6_ and ^5^S_2_/^5^F_4_ levels, respectively, leading to the loss of efficiency of the GRN emission (and also of the PL signal at around 750 nm) and to the corresponding increase in the population of the levels involved in the photophysical mechanism responsible for the RED emission.

**Figure 5:**
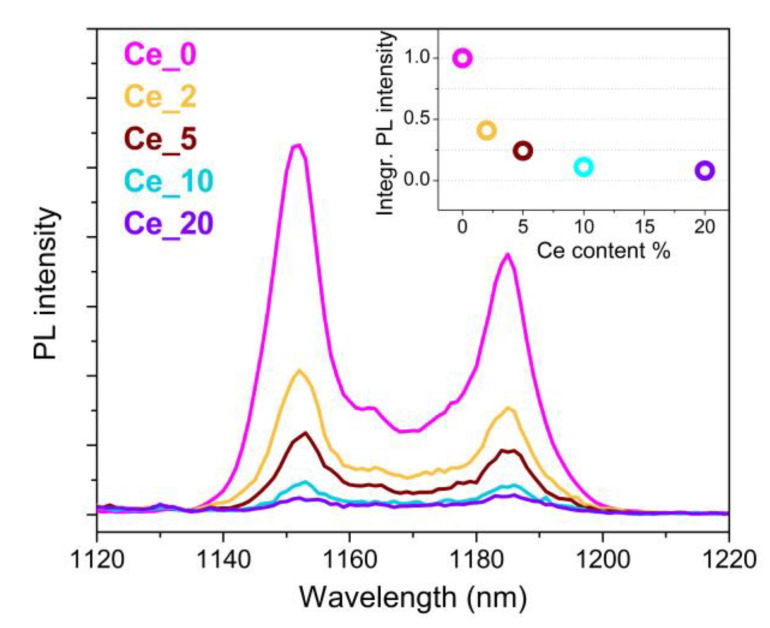
Ho^3+ 5^I_6_ → ^5^I_8_ PL spectra under 980 nm excitation of the Ln^3+^-doped NaBiF_4_ samples with different Ce^3+^ content. In the inset, trend of integrated PL intensity vs. Ce^3+^ content, with signals normalized to Ce_0 sample.

To account for the evolution of the Ho^3+ 5^I_6_ level occupancy, which is related to the $N_{2}^{Ho}$ parameter, Figure 5 shows the NIR emission deriving from Ho^3+ 5^I_6_ radiative relaxation to the ground state, as a function of the Ce^3+^ content. The fact that the luminescence signal promptly drops down as the Ce^3+^ content increases is a clear evidence of the Ho^3+ 5^I_6_ level depletion driven by the occurrence of the CR1 process.

For a quantitative assessment of the efficiency of the CR1 process, from Equation (10) we define the ratio of the NIR emission intensity due to Ho^3+ 5^I_6_ relaxation between a Ce^3+^-doped sample and the undoped reference as:

(14) $$\frac{I_{NIR}\left[\mathrm{Ce}\text{\_}X\right]}{I_{NIR}\left[\mathrm{Ce}\text{\_}0\right]}=\frac{w_{2}}{\left(w_{2}+c_{2}N_{0}^{Ce}\right)}\;.$$

The Ce^3+^ content dependence of this parameter can be evaluated from the trend of the experimental data shown in the inset of Figure 5.

The efficiency $\eta_{CR1}$ of the CR1 mechanism can be expressed as the ratio between the rate of the Ce^3+^-mediated process and the overall rate of the mechanisms that drive Ho^3+ 5^I_6_ depletion. Therefore, considering Equation (14), we obtain:

(15) $$\eta_{CR1}=\frac{c_{2}N_{0}^{Ce}}{\left(w_{2}+c_{2}N_{0}^{Ce}\right)}=1-\frac{I_{NIR}\left[\mathrm{Ce}\text{\_}X\right]}{I_{NIR}\left[\mathrm{Ce}\text{\_}0\right]}\;.$$

The results of the calculation for the different Ce^3+^-doped samples are reported in Table 1. It is worth noting that already with 2 at.% of Ce^3+^, the estimate is around 60% for $\eta_{CR1}$ parameter, which then grows to over 90% by increasing the Ce^3+^ content.

At this point, a comparison with the assessment of the efficiency for the CR2 process is proposed. In this case, the data for efficiency assessment have been extracted by considering the results of PL measurements by direct photoexcitation of Ho^3+^ ions under visible light exposure.

**Figure 6:**
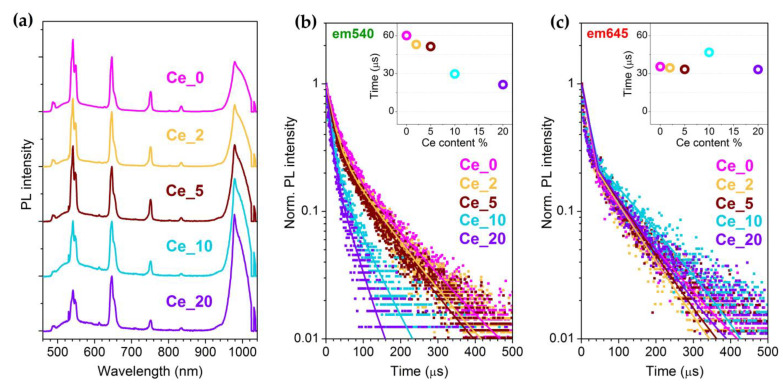
(**a**) PL spectra under 448 nm excitation of the Ln^3+^-doped NaBiF_4_ samples with different Ce^3+^ content; each spectrum is normalized to the RED band signal. (**b**,**c**) GRN and RED PL decay curves under 460 nm excitation of the Ln^3+^-doped NaBiF_4_ samples with different Ce^3+^ content; solid lines are the result of the double-exponential fit of the decay curves by using the function I(t)=I0+Ae−tτ1+Be−tτ2, where $\tau_{1}$ and $\tau_{2}$ represent the fast and the slow components of the overall decay process, respectively. In the inset, trend of GRN and RED lifetime τ parameters vs. Ce^3+^ content, where τ is determined through the weighted average calculation $\tau =\frac{A\tau_{1}+B\tau_{2}}{A+B}$.

The spectra of Figure 6a show the typical Ho^3+^ emission signals in the visible range, together with a band at around 980 nm referable to the excited-to-ground-state relaxation originating from Yb^3+ 2^F_5/2_ level. This aspect is addressed in the next section. Figure 6b,c report the time-resolved PL decay curves for the Ho^3+^ GRN and RED emissions. As can be inferred from the trend of the lifetime estimates reported in the two insets, the GRN values progressively decrease as the Ce^3+^ content increases, while the RED one remains substantially unchanged. The different behaviour is strictly linked to the role played by the CR2 mechanism, through which the presence of Ce^3+^ ions involves the activation of a further non-radiative de-excitation channel of Ho^3+ 5^S_2_/^5^F_4_, with consequent GRN emission weakening.

The CR2 conversion efficiency $\eta_{CR2}$ can be calculated according to the following equation, based on the lifetime ratio between a Ce^3+^-doped and the undoped sample:

(16) $$\eta_{CR2}=1-\frac{\tau_{GRN}\left[\mathrm{Ce}\text{\_}X\right]}{\tau_{GRN}\left[\mathrm{Ce}\text{\_}0\right]}\;.$$

The values obtained for the investigated samples are reported in Table 1 for a final comparison among the efficiencies of the two Ce^3+^-mediated CR processes. What emerges is that the $\eta_{CR1}$ parameter is always greater than $\eta_{CR2}$ in the whole explored Ce^3+^ doping range, and therefore, it can be stated that CR1 process plays a primary role in the observed GRN-to-RED conversion effect induced by Ce^3+^ incorporation in the NaBiF_4_:Yb^3+^/Ho^3+^ system.

### 3.5. Visible-to-NIR DC Effect

Here, we return to discuss the PL spectra in Figure 6a, obtained by direct Ho^3+^ excitation into ^5^F_1_/^5^G_6_ level through 448 nm pumping source. In addition to the typical visible emissions attributable to Ho^3+^ radiative transitions, a fluorescence band around 980 nm was also observed, which is linked to the Yb^3+ 2^F_5/2_ → ^2^F_7/2_ relaxation. As these are the only Yb^3+^ intra-4f transitions, it is reasonable to hypothesize that the activation of the 980 nm band originates from a mechanism of indirect Yb^3+^ excitation mediated by Ho^3+^ ions. Several studies have already investigated the peculiarities of this phenomenology, highlighting its potentiality for the conversion of high-energy radiation into NIR photons in view of applications in the field of photovoltaics and solar cells technology [42,43,44,45].

**Figure 7:**
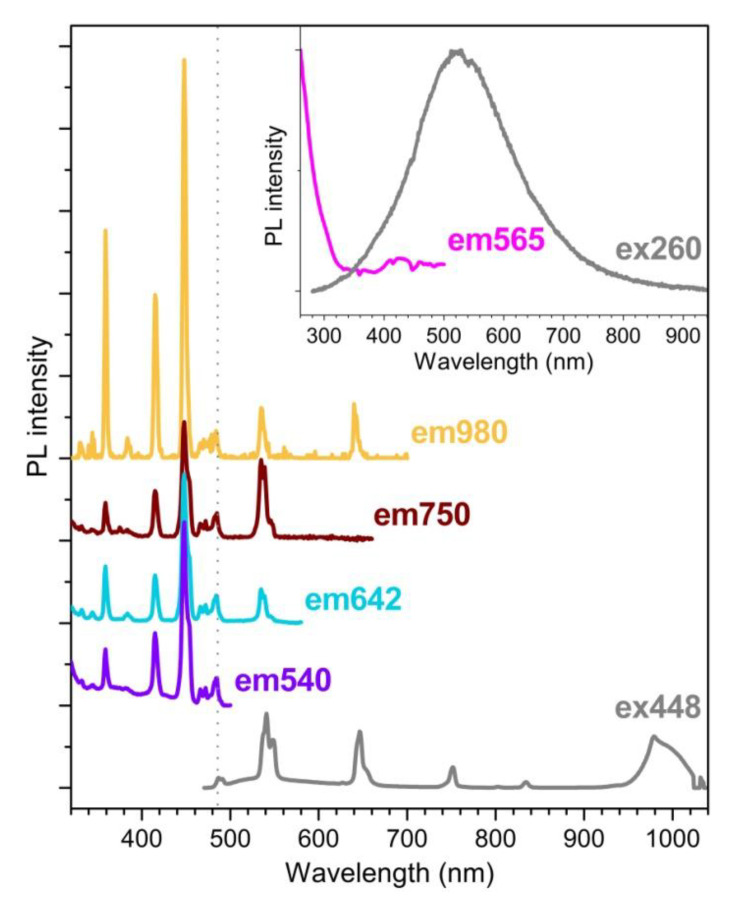
PLE spectra of emission signals at 540, 642, 750 and 980 nm and PL spectrum (grey line) under 448 excitation of Ce_0 sample; each PLE spectrum is normalized to the signal at 483 nm, corresponding to the Ho^3+ 5^I_8_ → ^5^F_3_ transition, which is marked by the vertical dotted line. In the inset, PLE spectrum of emission signal at 565 nm and PL spectrum under 260 nm excitation of Ce_0 sample.

Figure 7 reports a series of PLE spectra for the Yb^3+^/Ho^3+^ codoped sample (Ce_0), obtained by monitoring the intensity for the three main Ho^3+^ emissions in the visible range and the Yb^3+^ emission at 980 nm. To facilitate the comparison, the spectra were normalized with respect to the emission signal resulting under 483 nm excitation, corresponding to the Ho^3+ 5^I_8_ → ^5^F_3_ ground-to-excited-state absorption. This gives the possibility to ascertain that, while the PLE spectra for Ho^3+^ emissions are characterized by almost the same intensity ratio between the different peaks in resonance with Ho^3+^ absorptions, for the Yb^3+^ emission the feature at 483 nm marks a sort of threshold beyond which the higher-energy peaks show enhanced relative intensity. This suggests a larger efficiency of the visible-to-NIR DC for the Yb^3+^/Ho^3+^ codoped system when operating with excitation light towards the UV-visible edge.

**Figure 8:**
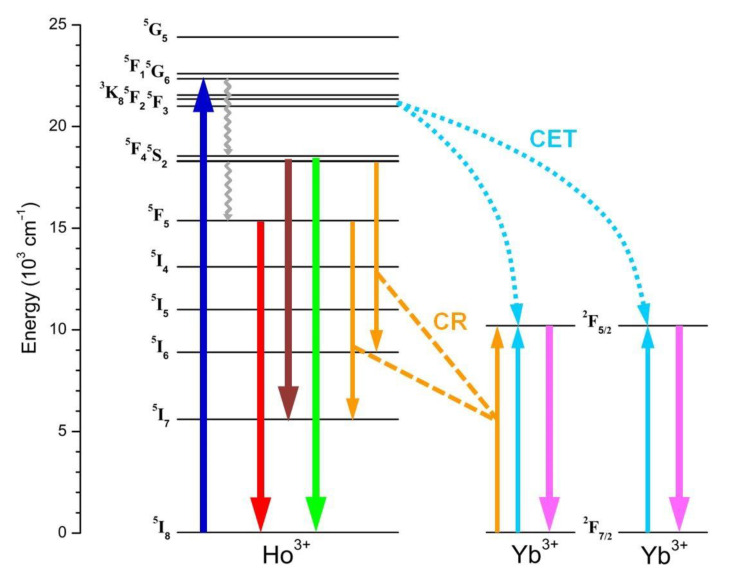
Scheme of the energy level diagram representing the photophysical mechanisms involved in the downconverted PL emissions observed for the Ln^3+^-doped NaBiF_4_ samples. The blue upward arrow refers to Ho^3+^ excitation into high-energy 4f levels; thick downward arrows refer to Ho^3+^ radiative relaxations in the visible range, and Yb^3+^ excited-state relaxation (purple arrow); cyan and orange arrows refer to Ho^3+^-Yb^3+^ cooperative energy transfer (CET) and cross-relaxation (CR) processes, respectively; grey wavelike arrows refer to multiphonon relaxations.

To get deeper inside the mechanism underlying the observed DC process, the scheme in Figure 8 depicts two of the major energy transfer processes proposed as responsible of the Ho^3+^-mediated Yb^3+^ ion photoexcitation [43]. On the one hand, upon Ho^3+^ excitation into high-energy 4f levels, which is followed by quick phonon-assisted relaxation to lower-lying ones responsible of Ho^3+^ visible emissions, resonant CR processes can take place, involving the transitions (i) Ho^3+ 5^S_2_/^5^F_4_ + Yb^3+ 2^F_7/2_ → Ho^3+ 5^I_6_ + Yb^3+ 2^F_5/2_ and (ii) Ho^3+ 5^F_5_ + Yb^3+ 2^F_7/2_ → Ho^3+ 5^I_7_ + Yb^3+ 2^F_5/2_. The observation in the 980 nm PLE spectrum of Figure 7 of specific features at the wavelengths of Ho^3+^ direct pumping into ^5^S_2_/^5^F_4_ and ^5^I_6_ levels could reflect the activation of these CR processes.

On the other hand, it has been proposed that, at high Yb^3+^ doping level, Ho^3+^-Yb^3+^ pairs can effectively interact through a cooperative energy transfer (CET) mechanism that originates from the Ho^3+^ levels involved in non-radiative relaxation processes, in particular, Ho^3+ 5^F_3_ with transition scheme Ho^3+ 5^F_3_ → 2Yb^3+ 2^F_5/2_, as it nearly falls at twice the energy for Yb^3+^ ground-state relaxation [43]. As this CET mechanism is configurable as a two-photon NIR QC and supposing an intrinsic larger efficiency with respect to the mentioned CR processes, this would explain the enhanced relative intensity of the PLE peaks in the 980 nm spectrum when pumping with photons in the UV-blue range.

A further aspect to note is linked to a possible role of Ce^3+^ in the observed visible-to-NIR DC process. The PL spectra of Figure 6a show that the increase in Ce^3+^ content determines an enhancement of the Yb^3+^ 980 nm band with respect to the different Ho^3+^ emissions in the visible range. This cannot be solely attributed to a Ce^3+^-induced loss in efficiency for the Ho^3+^ radiative processes, otherwise we would expect at least a lifetime decrease for the Ho^3+^ PL emission at 650 nm. Rather, the idea is that the presence of Ce^3+^ can foster the Ho^3+^-Yb^3+^ ET coupling. In this regard, in the literature, a role of the Ce^3+^ 5d states is invoked, suggesting a first ET process from Ho^3+^ high-energy 4f levels to Ce^3+^ 5d ones upon UV-blue light pumping, followed by a Ce^3+^-Yb^3+^ cooperative DC involving Ce^3+^ 5d non-radiative relaxation and ET to a pair of Yb^3+^ ions, with final 980 nm photon emission [46].

As a final suggestion, it is worth considering a peculiar aspect that emerges in the PL spectra of Figure 6a. These show that the Ho^3+^ emission bands in the visible range seem to float above a background luminescence signal. The inset of Figure 7 highlights that, under UV photoexcitation, this PL signal is strongly enhanced, resulting in a wide band with peak located at around 565 nm and characterized by a large Stoke shift with respect to the 300 nm absorption threshold which appears in the PLE spectrum. Luminescence emissions in the visible spectral range, also active at room temperature, have been observed for the NaBiF_4_ system [38]. In general, RT luminescence activity observed for bismuth-based compounds is typically attributed to Bi^3+^ s-p transitions.

## 4. Conclusions

This work has focused on the investigation of the luminescence properties exhibited by NaBiF_4_ NPs embedding specific amount of Yb^3+^, Ho^3+^ and Ce^3+^ ions. Operating in UC mode, which determines NIR-to-visible conversion through Yb^3+^-Ho^3+^ ET mechanisms, we observed that the variation of the Ce^3+^ content induces a modification of the PL chromaticity output as determined from the relative intensity of the two main visible Ho^3+^ emission features (GRN and RED bands). The effect is attributed to a pair of efficient CR mechanisms, by which a color tuning effect takes place with progressive green-to-red conversion with increasing Ce^3+^ content. On the basis of a rate equations model, we established a hierarchy between the two Ce^3+^-mediated CR processes from the experimental data, and a parabolic dependence on the Ce^3+^ content of the intensity ratio between the Ho^3+^ RED and GRN emissions in a specific Ce^3+^ doping range, beyond which a saturation effect is observed.

Furthermore, direct photoexcitation of Ho^3+^ ions into high-energy levels originates luminescence spectra featuring an intense 980 nm Yb^3+^ band as the result of a visible-to-NIR DC mechanism. The Yb^3+^ emission process is particularly efficient with UV-visible pumping above the energy threshold corresponding to the Ho^3+ 5^I_8_ → ^5^F_3_ transition, then suggesting the occurrence of a QC process driven by Ho^3+^-Yb^3+^ CET that enhances the overall DC mechanism. Moreover, we observed that the presence of Ce^3+^ ions can contribute to this process by further fostering the visible-to-NIR DC effect.

All these evidences make Yb^3+^/Ho^3+^/Ce^3+^ doping of the developed NaBiF_4_ NPs as a promising way to prepare phosphors with controlled UC and DC emission performance for possible applications in several fields such as photonics, bioimaging and anticounterfeiting.

## Figures and Tables

**Table 1:** Results of the intensity measurements of Ho^3+^ NIR emission, and of the calculations related to Ho^3+^ GRN emission lifetime and Ce^3+^-mediated CR efficiency for the investigated Ln^3+^-doped NaBiF_4_ samples.

Sample	$I_{NIR}$	$\eta_{CR1}$	$\tau_{GRN}$	$\;\eta_{CR2}$
	*normalized to Ce_0*	[%]	[ms]	[%]
Ce_0	1.000	-	60.1	-
Ce_2	0.410	59.0	53.1	11.7
Ce_5	0.300	75.8	51.5	14.3
Ce_10	0.137	88.9	29.7	50.5
Ce_20	0.101	91.8	21.3	64.6

## Data Availability

The data presented in this study are available on request from the corresponding author.

## Supplementary Materials

The following supporting information can be downloaded at: https://www.mdpi.com/article/10.3390/nano13040672/s1, Figure S1: Trend of cell volume vs. Ce^3+^ content; Figure S2: FE-SEM images of Ce_0, Ce_2, Ce_5 and Ce_20 samples; Figure S3: EDS spectrum of Ce_10 sample; Figure S4: FE-SEM image and elemental maps of Ce_10 sample.
