# Supplementary material for: Light Conversion upon Photoexcitation of NaBiF4:Yb3+/Ho3+/Ce3+ Nanocrystalline Particles
Source: Nanomaterials (Basel). 2023 Feb 9;13(4):672. doi: 10.3390/nano13040672 (PMC9963621; doi:10.3390/nano13040672)
Supplement: Supplementary file 1 [file nanomaterials-13-00672-s001.zip › nanomaterials-2159934-supplementary.pdf]

Light Conversion upon Photoexcitation of $NaBiF_4:Yb^{3+}/Ho^{3+}/Ce^{3+}$ Nanocrystalline Particles

Enrico Trave [1,*], Michele Back [1,*], Davide Pollon [1], Emmanuele Ambrosi [1] and Leonardo Puppulin [1,2]

[1] Department of Molecular Sciences and Nanosystems, Università Ca' Foscari Venezia, Via Torino 155, 30172 Venice, Italy; pollon.davide@gmail.com (D.P.); emmanuele.kizito@gmail.com (E.A.); leonardo.puppulin@unive.it (L.P.)

[2] WPI Nano Life Science Institute (WPI-NanoLSI), Kanazawa University, Kanazawa, Ishikawa 920-1192, Japan

* Correspondence: enrico.trave@unive.it (E.T.); michele.back@unive.it (M.B.)

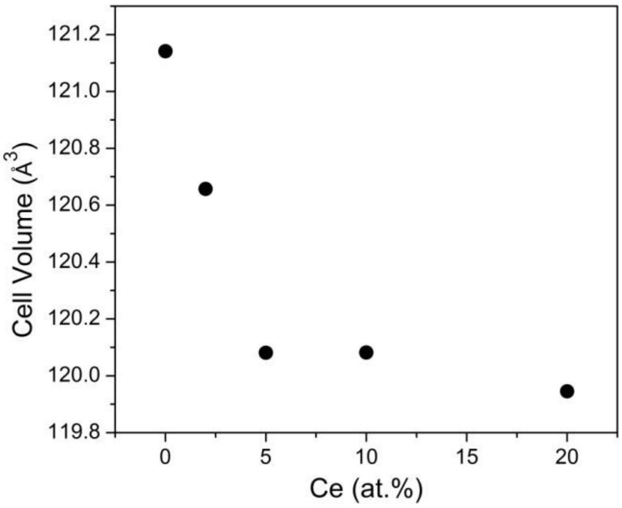

Figure S1. Trend of cell volume vs. Ce^{3+} content; cell volume estimation was obtained from fitting of XRPD patterns reported in Figure 1(a).

Citation: Trave, E.; Back, M.; Pollon, D.; Ambrosi, E.; Puppulin, L. Light Conversion upon Photoexcitation of $NaBiF_4:Yb^{3+}/Ho^{3+}/Ce^{3+}$ Nanocrystalline Particles. *Nanomaterials* **2023**, *13*, 672. https://doi.org/10.3390/nano13040672

Academic Editors: Vincenzo Vaiano

Received: 29 December 2022
Revised: 1 February 2023
Accepted: 5 February 2023
Published: 9 February 2023

Copyright: © 2023 by the authors. Licensee MDPI, Basel, Switzerland. This article is an open access article distributed under the terms and conditions of the Creative Commons Attribution (CC BY) license (https://creativecommons.org/licenses/by/4.0/).

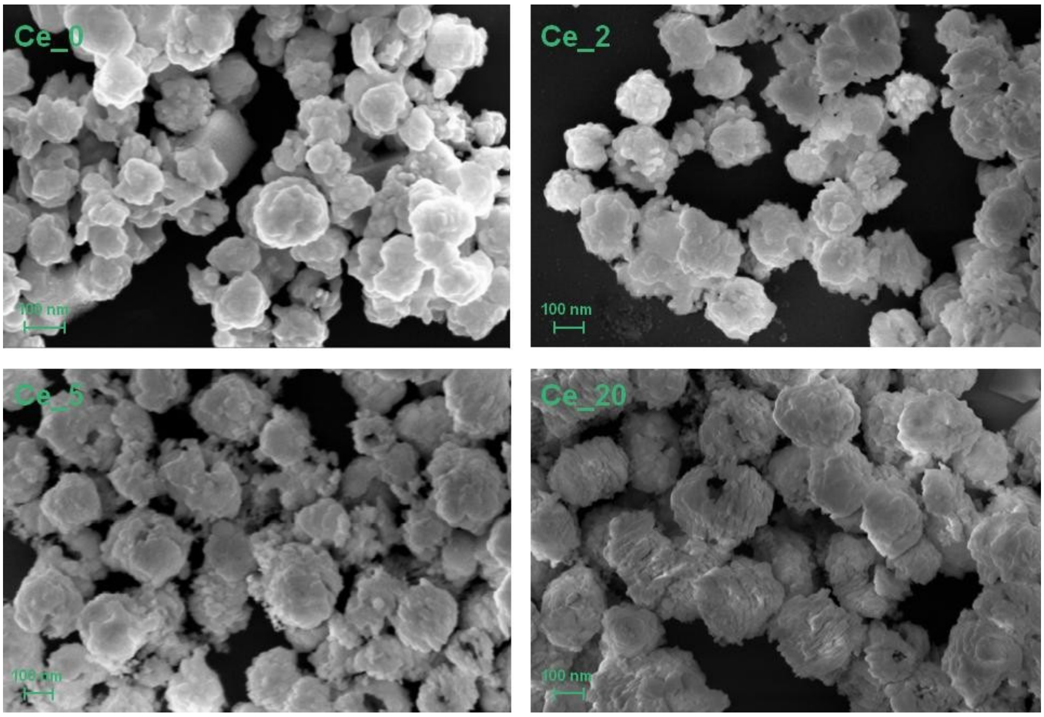

Figure S2. FE-SEM images of Ce_0, Ce_2, Ce_5 and Ce_20 samples.

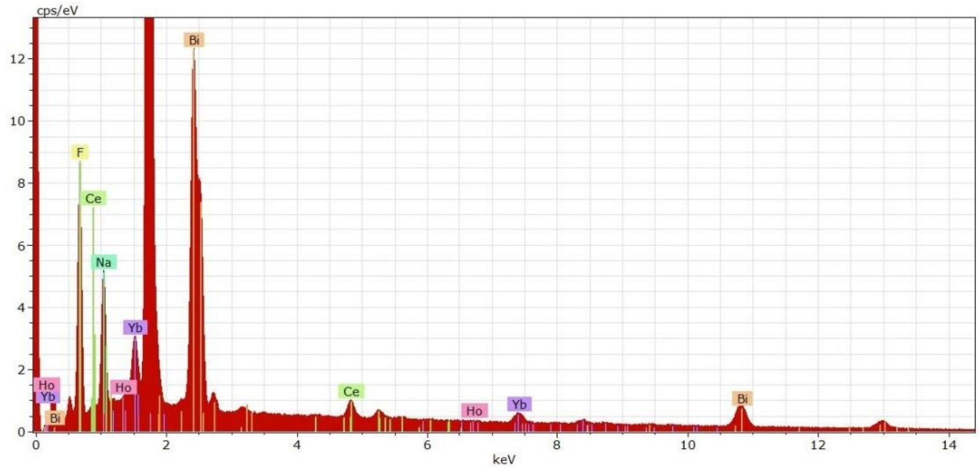

Figure S3. EDS spectrum of Ce_10 sample.

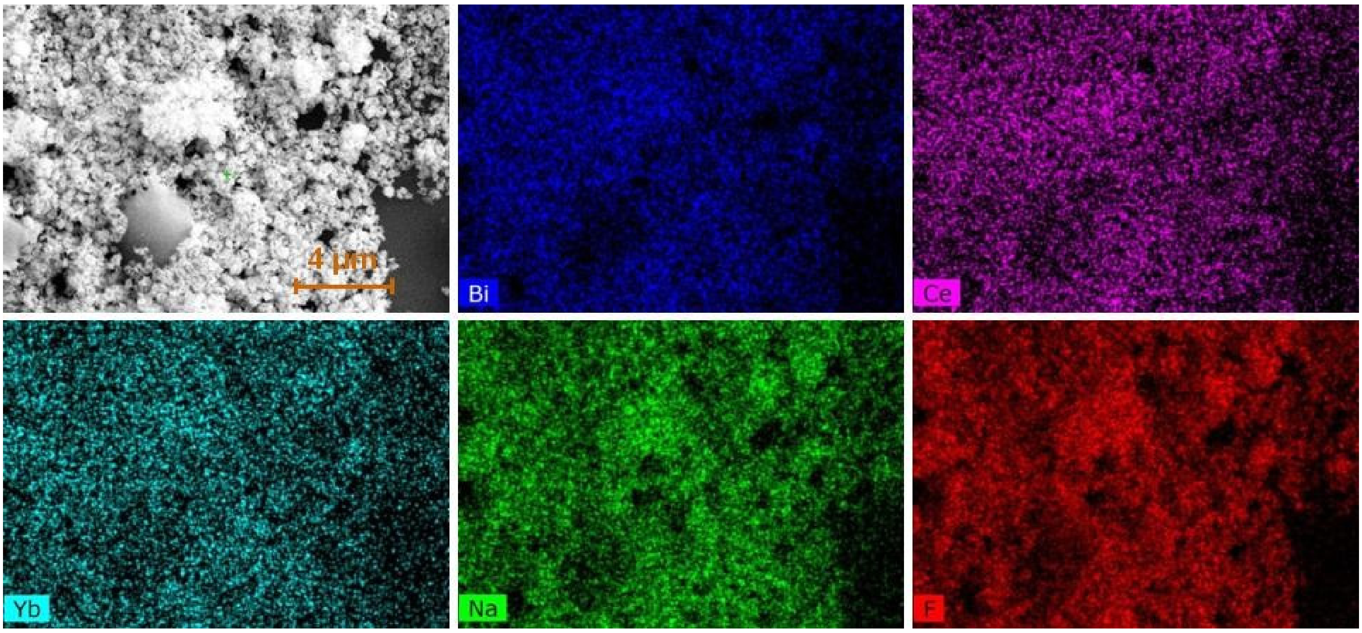

Figure S4. FE-SEM image and elemental maps of Ce_10 sample.
